# Proximal Tubule p53 in Cold Storage/Transplantation-Associated Kidney Injury and Renal Graft Dysfunction

**DOI:** 10.3389/fmed.2021.746346

**Published:** 2021-10-22

**Authors:** Xiaohong Xiang, Jiefu Zhu, Gang Zhang, Zhengwei Ma, Man J. Livingston, Zheng Dong

**Affiliations:** ^1^Department of Nephrology, Hunan Key Laboratory of Kidney Disease and Blood Purification, The Second Xiangya Hospital of Central South University, Changsha, China; ^2^Department of Cellular Biology and Anatomy, Medical College of Georgia at Augusta University and Charlie Norwood Veterans Affairs Medical Center, Augusta, GA, United States; ^3^Center of Nephrology and Dialysis, Transplantation, Renmin Hospital of Wuhan University, Wuhan, China; ^4^Center of Organ Transplantation, Xiangya Hospital, Central South University, Changsha, China

**Keywords:** cold storage, kidney injury, kidney transplantation, p53, proximal tubule

## Abstract

Kidney injury associated with cold storage/transplantation is a primary factor for delayed graft function and poor outcome of renal transplants. p53 contributes to both ischemic and nephrotoxic kidney injury, but its involvement in kidney cold storage/transplantation is unclear. Here, we report that p53 in kidney proximal tubules plays a critical role in cold storage/transplantation kidney injury and inhibition of p53 can effectively improve the histology and function of transplanted kidneys. In a mouse kidney cold storage/transplantation model, we detected p53 accumulation in proximal tubules in a cold storage time-dependent manner, which correlated with tubular injury and cell death. Pifithrin-α, a pharmacologic p53 inhibitor, could reduce acute tubular injury, apoptosis and inflammation at 24 h after cold storage/transplantation. Similar effects were shown by the ablation of p53 from proximal tubule cells. Notably, pifithrin-α also ameliorated kidney injury and improved the function of transplanted kidneys in 6 days when it became the sole life-supporting kidney in recipient mice. *in vitro*, cold storage followed by rewarming induced cell death in cultured proximal tubule cells, which was accompanied by p53 activation and suppressed by pifithrin-α and dominant-negative p53. Together, these results support a pathogenic role of p53 in cold storage/transplantation kidney injury and demonstrate the therapeutic potential of p53 inhibitors.

## Introduction

Kidney transplantation is a preferred treatment for end stage renal disease (ESRD) for its advantage in better life quality and lower medical cost. However, the demand of optimal living donor kidneys has far exceeded the availability. As a result, deceased donors remain to be a major source for kidney transplants (1). From deceased donors to recipients, the kidneys are inevitably subjected to a series of ischemia and hypothermia; reperfusion and rewarming injury (2). Particularly, the preservation and transportation of these deceased donor kidneys are often associated with cold ischemia, which has been shown clearly to be an independent risk factor for delayed graft function (DGF) and chronic nephropathy (3, 4). Strategies and approaches to alleviate cold storage/transplantation injury would improve the quality of the donor kidneys and set stage for greater acceptance of the grafts. In cold storage/transplantation, significant injury and cell death occurs in renal tubules, especially the proximal tubules, contributing to compromised graft function and survival (5). Basic research (6, 7) and clinical trial (8) revealed that inhibition of proximal tubular cell death with caspase inhibitors during cold storage improves graft function and histology. Moreover, we (9) and others (10–13) identified intrinsic mitochondrial pathway of apoptosis in cold storage/transplantation kidney injury. Despite these findings, the cellular and molecular mechanism of cold storage/transplantation kidney injury remains elusive and there is an urgent need for effective renoprotective approaches.

p53, a well-known tumor suppressor, is a stress response gene (14). In kidneys, p53 contributes to the pathogenesis of both acute and chronic kidney diseases (15, 16). In 2003, the Dagher lab demonstrated the protective effect of pifithrin-α in renal ischemia-reperfusion, suggesting the first evidence for a role of p53 in acute kidney injury (17). In 2004, we suggested the role of p53 in cisplatin-induced kidney injury (18). Later studies by us and the Padanilam lab demonstrated that knockout of p53 from proximal tubules alleviates ischemic kidney injury in mice, providing unequivocal evidence for the pathogenic role of p53 in AKI (19, 20). In view of these studies, the current study was designed to (1) delineate p53 activation in kidney cold storage/transplantation, (2) elucidate the role of p53 in cold storage/transplantation kidney injury, (3) identify the specific role of proximal tubule p53, and (4) test the therapeutic potential of p53 inhibitors in kidney cold storage/transplantation.

## Materials and Methods

### Animals

Animals used in this study were housed in the animal facility of Charlie Norwood Veterans Affairs Medical Center (CNVAMC). Animal experiments were conducted with the approval of and in accordance with the guidelines established by the Institutional Animal Care and Use Committee of CNVAMC. Male mice on C57BL/6 background of 8–14 weeks of age were used. The kidney proximal tubule-specific p53 knockout mouse model was generated by crossing p53-floxed mice with PEPCK-Cre mice as described previously (19).

### Kidney Cold Storage/Transplantation

The kidneys from donor mice were transplanted into syngeneic recipients following previously described procedures with minor modifications (9, 21). The donor mice were anesthetized with pentobarbital (50 mg/kg intraperitoneally). The left kidney of the donor was excised and flushed with cold heparinized (Sigma H3393) University of Wisconsin (UW) solution (Belzer UW; Bridge to Life Ltd.) and stored in cold UW solution in ice-water bath for the indicated time. For recipient transplantation, the left kidney of the recipient was removed. The donor aortic cuff and renal vein were anastomosed to the recipient abdominal aorta and inferior vena cava, respectively, with 10-0 Ethicon sutures. Graft anastomosis time was standardized at 30 min. The ureteral implantation was accomplished by fixing to the exterior wall of the bladder. The end of the ureter was cut and the bladder wall was closed using a 10-0 Ethicon suture. During the recipient surgery, the animals were kept hydrated with normal sterilized saline instilled intraperitoneally. During surgery, the mice were kept on warm heating pads to maintain body temperature. After transplantation, recipient mice were euthanized at 24 h to collect transplanted kidneys for analysis of acute injury. To monitor the life-supporting function of transplanted kidneys, the native contralateral kidney was removed at day 5 post-transplantation to let the mice survive for another day to examine the function and histology of the transplanted kidney at day 6.

To determine the effect of pifithrin-α (Selleck, S2929) on acute kidney cold storage/transplantation injury, it was given three times: (1). Injected to donor mice overnight before donor kidney procurement; (2). Kept in UW solution at 50 μM during cold preservation according to cell experiment; (3). added to abdominal cavity immediately after transplantation (day 0) to recipient mice. To test its effect in the life supporting model, in addition to those three dosages, pifithrin-α was given two more dosages on day 2 and day 4 after transplantation. All the injection dosages were intraperitoneally administered at 2.2 mg/kg. The same volume of DMSO (the vehicle solution) was given as the control of every dosage. Purity of the pifithrin-α was shown to be 98.14% by the manufacturer (Selleck. TX) in HPLC assay.

Renal function was measured as serum creatinine and blood urine nitrogen (BUN) levels using commercial kits as previously described (22).

### Cold Storage/Rewarming of Rat Kidney Proximal Tubular Cells

Rat kidney proximal tubule cells (RPTCs) were originally from U. Hopfer at Case Western Reserve University (Cleveland, OH) and cultured as described previously (18). For cold storage, cells were incubated in UW solution at 4°C for indicated time. For rewarming, UW solution was replaced with normal culture medium at 37°C for indicated time. Cell death was evaluated by phase-contrast and fluorescence microscopy after stained with Hoechst 33342. Cells showing cellular shrinkage, blebbing, and nuclear condensation and fragmentation were counted as apoptotic cells. The percentage of cell apoptosis was estimated in four fields with ~200 cells per field to estimate the percentage of apoptosis.

### Stable Transfection of RPTCs With Dominant Negative p53

RPTCs stably transfected with dominant-negative p53 were generated in our previous work (18). Briefly, the dominant-negative mutant sequence of p53 with a point mutation (V143A) (a generous gift from Dr. Chi-Hui Tang, University of Texas M.D Anderson Cancer Center and Health Science Center, Houston, TX) was subcloned into pcDNA3.1 that contained a HA tag and hygromycin resistance cassette using *Hind*III and *Xba*I. After transfection using Lipofectamin 2000 (*Invitrogen*), the cells were maintained in culture medium containing hygromycin for selection and expanding. Positive clones verified by immunoblotting of HA were subjected to a second round of cloning to ensure the selection of pure colonies.

### Determination of Caspase Activity

Caspase activity was measured by the enzymatic assay using the fluorogenic peptide substrate DEVD.AFC as described previously (23). The 1% Triton X-100 extracted cell lysate was added to the enzymatic reaction containing DEVD.AFC. The fluorescence at excitation 360 nm/emission 530 nm was monitored by a GENios plate-reader (Tecan US Inc., Research Triangle Park, NC, USA). A standard curve was constructed using free AFC in each measurement. Caspase activity was expressed as the nanomolar amount of liberated AFC by each mg protein of cell lysate.

### Immunoblot Analysis

Immunoblotting was performed by standard method as previously described (24). The primary antibodies used in this study included p53 (#2524), p-p53 (s15) (#9284, #9286), PARP (#9532), cleaved caspase-3 (#9664), Bak (#12105), PUMA (#4976), HA tag (#5017), cyclophilin B (#43603) and GAPDH (#5174) from Cell signaling, and p21 (ahz0422) from *Invitrogen*. Each experiment was repeated for at least four times, and representative images are present.

### Hematoxylin and Eosin Staining and Terminal Deoxynucleotidyl Transferase–Mediated Digoxigenin-Deoxyuridine Nick-End Labeling (TUNEL) Assay

For histology, kidney tissues were fixed for paraffin embedding and H&E staining. Tubular damage was scored by the percentage of renal tubules with cell lysis, loss of brush border, and cast formation (0, no damage; 1, <25%; 2, 25–50%; 3, 50–75%; 4, >75%) (25). For TUNEL staining, kidney tissue sections were stained with *In Situ* Cell Death Detection Kit, TMR red (Roche Applied Science) and examined with fluorescent microscopy. The slides were examined with fluorescent microscopy, and the TUNEL-positive cells were counted from 10 randomly picked images for each specimen in the outer medulla and kidney cortex region.

### Immunofluorescence of Tissue and RPTCs

Kidney samples were fixed, paraffined, rehydrated for immunofluorescence staining with procedure as previously described (24). The primary antibodies included anti-p53 (#2524) and anti-p-p53 (s15) (#9286) from Cell Signaling. Fluorescein-labeled Lotus Tetragonolobus Lectin (LTL, FL-1321; Vector Labs) or DAPI (Vector) was used to mark proximal tubules or nucleus, respectively. For quantification, positive cells were counted from ten randomly picked images for each specimen in the outer medulla and kidney cortex region. Proximal tubules were also stained with fluorescein-labeled LTL alone. For quantitative analysis, around 10 fields (X100 magnification) were randomly selected from each section and the percentage of normal LTL- positive tubule was measured with ImageJ.

RPTCs immunofluorescence was performed as described previously (26) using p53 antibody (#2524). Slides were mounted with anti-fade containing DAPI.

### Immunohistochemistry

Immunohistochemistry staining for KIM-1 was performed as previously described (9). Tissue was exposed to goat KIM-1 antibody (Novus, AF1750) and ImmPRESS-AP (Alkaline Phosphatase) horse anti-goat IgG. The color was developed using vector red substate kit, Alkaline phosphatase (AP) (SK-5100). For quantification, around 10 fields (X100 magnification) were randomly selected from each section and the percentage of KIM-1 positive tubules was measured with ImageJ.

Immunohistochemistry staining for macrophage and neutrophil used an amplification protocol as formerly described (24). After primary antibody incubation with rat anti-macrophage (ab56297, Abcam) and rat anti-neutrophil (ab2557, Abcam), kidney tissue sections were blocked with avidin and biotin (vector laboratory kit, SP-2001) followed by biotinylated goat anti-rat secondary antibody (Millipore, AP183B). Following signal amplification with Tyramide Signal Amplification Biotin System (Perkin Elmer, NEL700A001KT), the sections were incubated with a VECTAS-TAIN® ABC kit (Vector Laboratories, PK-6100) and color was developed with a DAB kit (Vector Laboratories). For quantitative analysis, 10 to 20 fields (X200 magnification) were randomly selected from each section and the percentage of positive stained area was quantitated using ImageJ.

### Statistical Analyses

The Student's t test was used to show the significant difference between two groups, and ANOVA was used for multigroup comparison. The Dunn's multiple comparisons and the Fisher least significant difference test were used for one-way ANOVA and two-way ANOVA, respectively. Linear regression analysis was performed using Prism. *P* values <0.05 were considered significant. Data were expressed as mean ± SD. GraphPad Prism 8 was used for all calculations.

## Results

### p53 Is Activated in kidney Transplantation After Cold Storage

**Figure 1:**
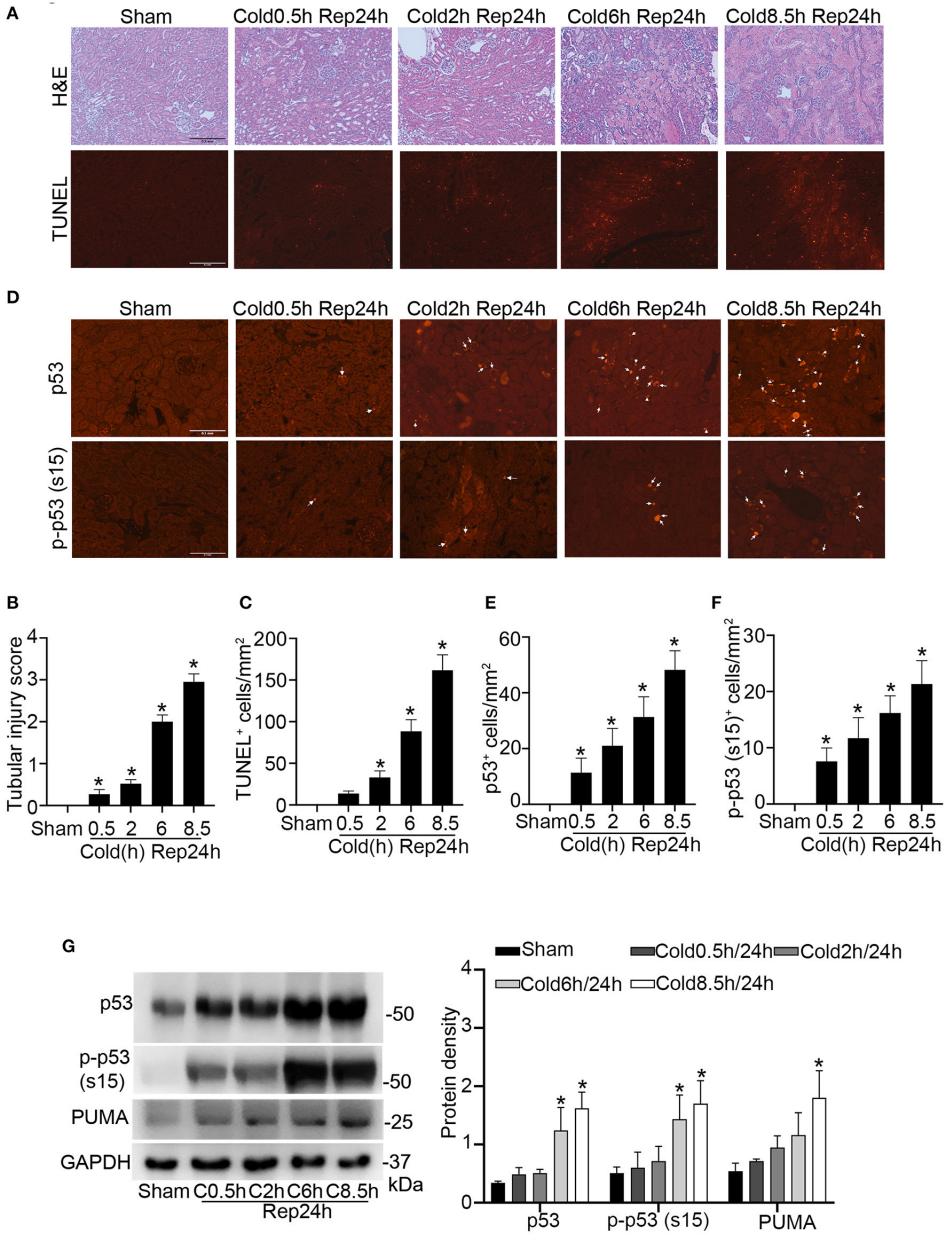
Kidney injury and p53 activation during cold storage/transplantation. The left kidney was collected from B6 donor mice for 0.5–8.5 h of cold storage, followed by transplantation into syngeneic recipient mice for 24 h or sham operation as control. **(A)** Representative images of H&E staining and TUNEL staining. Scale bars = 0.2 mm. **(B)** Pathological score of tissue damage. **(C)** Quantification of TUNEL positive cells in kidney cortical and outer medulla tissues. **(D)** Representative images of p53 and p-p53 (s15) immunofluorescence. Scale bars = 0.1 mm. Arrow: positive staining. **(E,F)** Quantification of p53 and p-p53 (s15) positive cells in kidney cortical and outer medulla tissues. **(G)** Immunoblots analysis and quantification of p53, p-p53 (s15), and PUMA. GAPDH was used as loading control. Data are expressed as means ± SD; n ≥ 4. *P < 0.05, statistically significant difference from the sham control group.

To study kidney cold storage/transplantation, we used a mouse syngeneic kidney transplantation model, in which the left kidney was isolated from the donor B6 mouse for cold storage and then transplanted to the recipient B6 mouse. In histology (Figure 1A), kidney transplantation with short durations of cold storage of 0.5 and 2 h only showed injury in a few tubules. Six h of cold storage caused moderate (scored ~2) while 8.5 h led to severe tubular damage (scored ~3) (Figure 1B), which was characterized by tubular brush border shedding, cast formation, cellular necrosis and detachment from basement membranes. Corroborating with the histological observations, tubular cell death detected by TUNEL assay (Figure 1A) slightly increased with short cold storage followed by transplantation. However, TUNEL positive cells increased significantly after 6 h, and peaked at 8.5 h, of cold storage with transplantation (Figure 1C). In this model, we initially examined p53 by immunofluorescence. As shown in Figure 1D, kidney cold-storage/transplantation increased p53-positive cells. Consistently, kidney cold-storage/transplantation led to p53 phosphorylation at serine-15 [p-p53 (s15)], indicative of p53 activation. In quantification, 0.5 and 2 h of cold storage with transplantation moderately increased the number of p53-positive cells, while 6 and 8.5 h induced remarkable increases (Figure 1E). Along with total p53, the phosphorylated p53 (p-p53) increased albeit at a lower level in quantification (Figures 1D,F). By co-staining with LTL (marker for kidney proximal tubules) and DAPI, we detected p53 and p-p53 (s15) mainly in the nucleus of proximal tubule cells although occasionally observed p53 and p-p53 (s15) staining in distal tubules and interstitium (Supplementary Figure 1). Immunoblot analysis of kidney tissues was roughly consistent with the cold-storage time dependent increases of p53, p-p53 (s15) and its downstream gene like PUMA (Figure 1G).

### p53 Activation Correlates With Cold-Storage Time and Tubular Injury in Transplanted Kidneys

**Figure 2:**
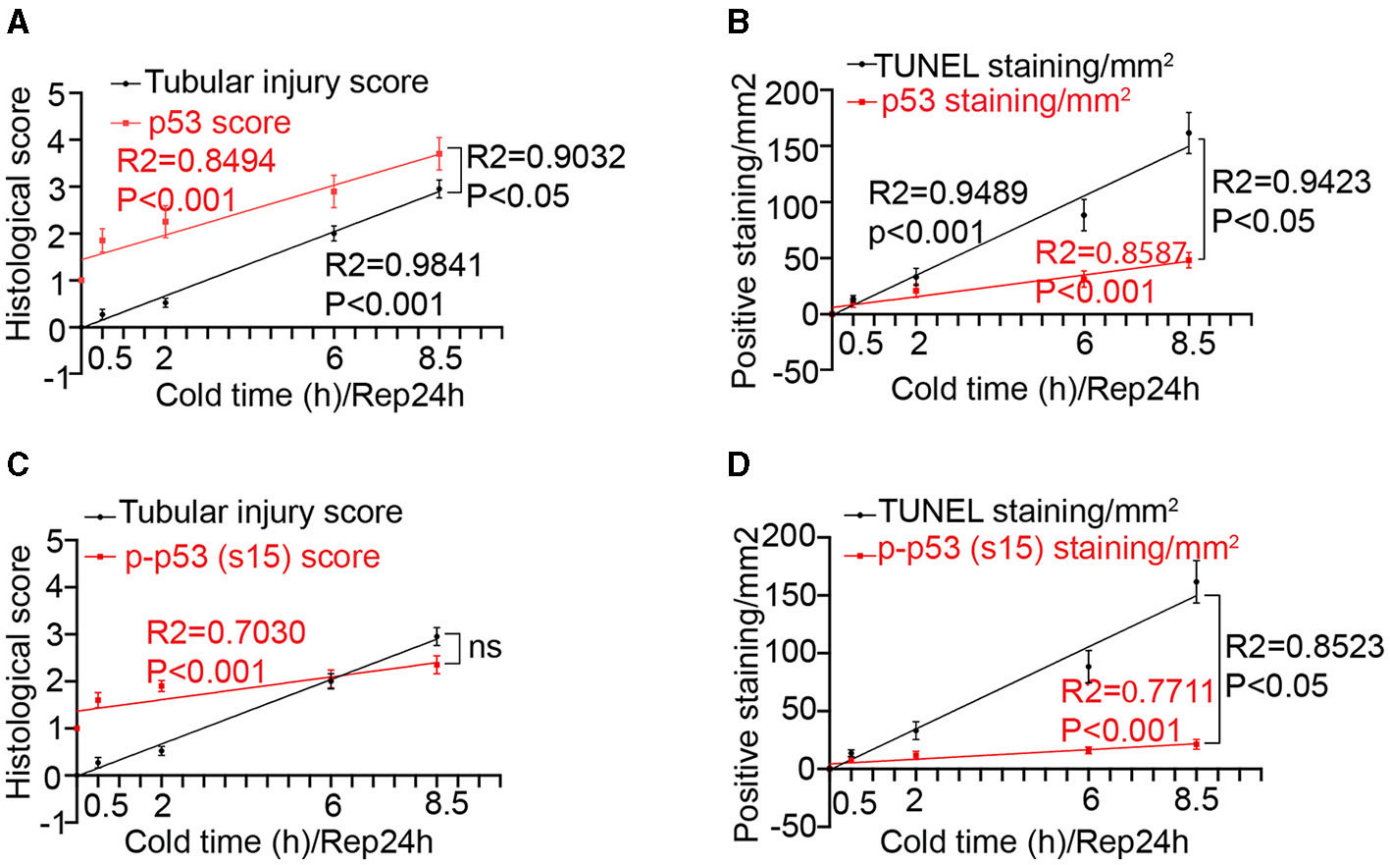
Correlations between cold-storage time, tubular injury and p53 in transplanted kidneys. **(A)** Linear correlation and regression analysis of p53 staining score and tubular injury score with cold storage time. **(B)** Linear correlation and regression analysis of p53 staining and TUNEL staining with cold storage time. **(C)** Linear correlation and regression analysis of p-p53 (s15) staining score and tubular injury score with cold storage time. **(D)** Linear correlation and regression analysis of p-p53 (s15) staining and TUNEL staining with cold storage time. Data are expressed as means ± SD; n ≥ 4.

We then conducted Pearson correlation and linear regression analysis to determine the relationships between p53 and tubular injury during kidney cold storage/transplantation. For the sake of analysis, we firstly determined the histological score of p53 and p-p53 (s15) staining on the basis of the average number of p53/p-p53 (s15)-positive cells per image in ×200 magnification according to our recent work (24): 1, no positive tubules; 2, 1 to 3; 3, 4 to 6; 4, 7 to 9; 5, 10 to 12; 6, 13 to 15; 7, 16 to 18; and 8, 19 to 21 positive tubules, respectively. As shown in Figure 2A, cold storage time correlated well with tubular injury (*R2* = 0.9841 *P* < 0.001) and p53 activation (*R2* = 0.8494 *p* < 0.001). Notably, tubular injury showed a good correlation with p53 activation with an R2 value of 0.9032. To reveal the association between p53 and cell death, the correlation between p53 staining and TUNEL staining was analyzed (Figure 2B). Cold storage time (*R2* = 0.9489 *P* < 0.001) as well as p53 activation (*R2* = 0.9423 P < 0.05) showed significant positive correlations with TUNEL positive staining. Furthermore, p-p53 (s15) correlated well with cold storage time (*R2* = 0.7030, *P* < 0.001 Figure 2C) and TUNEL staining (*R2* = 0.8523, *P* < 0.05, Figure 2D), but it did not show a significant correlation with renal tubular injury (Figure 2C).

### Pifithrin-α Inhibits p53 and Protects Renal Tubular Cells During Cold Storage/Rewarming

For *in vitro* experiments, we took advantage of cold storage/rewarming model to mimic the ATP-depletion and replenishment *in vivo* (9, 11, 27, 28). Rat kidney proximal tubular cells (RPTCs) were cold (4°C) stored in UW solution followed by incubation in full culture medium at 37°C for rewarming. Immunoblot analysis showed that p53, p-p53 (s15) and downstream targets gene (e. g., Bak and p21) were upregulated during rewarming after 24 h of cold storage, while cold storage only without rewarming did not induce significant changes in these proteins (Supplementary Figure 2).

**Figure 3:**
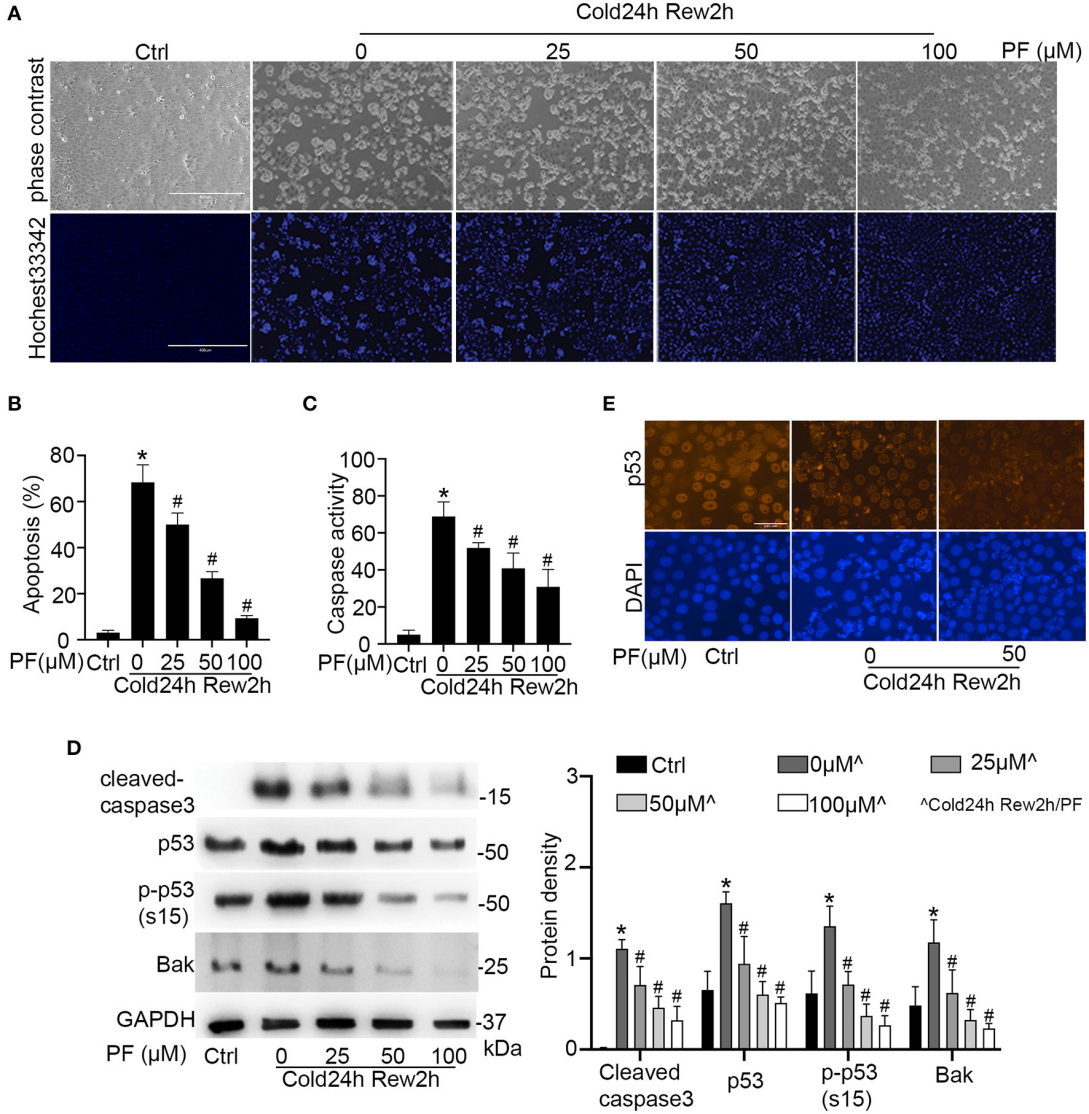
Pifithrin-α inhibits p53 and protects renal tubular cells during cold storage/rewarming. RPTCs were incubated in UW solution in 4°C for 24 h followed by rewarming in full medium in 37°C for 2 h in the presence of 0, 25, 50, or 100 μM pifithrin-α (PF). **(A)** Representative images of cellular and nuclear morphology. Cell nucleus was stained with Hochest 33342. Scale bar: 0.4 mm **(B)** Percentage of cell death quantified by counting the cells with typical apoptotic morphology. **(C)** Caspase activity measured with DEVD.AFC as the enzymatic substrate. **(D)** Immunoblots analysis and quantification of cleaved-caspase3, p53, p-p53 (s15) and Bak with GAPDH as a loading control. **(E)** Immunofluorescence of p53. DAPI was used to mark nucleus. Scale bar, 0.05 mm. Data are expressed as mean ± SD (n≥ 5). *P < 0.05 vs. Control. ^#^*P* < 0.05 vs. PF 0 μM (Cold 24 h Rew2h).

To determine the role of p53 in cold storage/rewarming-associated cell injury, we tested the effects of pifithrin-α, a pharmacological inhibitor of p53 (29). Cold storage only without rewarming did not induce obvious apoptosis in RPTCs (data not shown). Two h rewarming after 24 h of cold storage induced 68% apoptosis that was characterized by nuclear shrink, fragmentation and cell detach (Figure 3A). Propidium iodide (PI) staining did not show significant necrosis during cold storage/rewarming (data not shown). Pifithrin-α suppressed apoptosis during cold storage/rewarming dose-dependently. While 25 μM of pifithrin-α reduced apoptosis from 68 to 50%, 50 and 100 μM of pifithrin-α suppressed it to 27 and 10% respectively (Figures 3A,B). The morphological analysis was verified by measurement of caspase activity (Figure 3C) and by immunoblotting of active/cleaved caspase 3 (Figure 3D). The inhibitory effect of pifithrin-α on p53 was confirmed by immunoblot analysis of p53, p-p53 (s15) and its target gene Bak (Figure 3D) and immunofluorescence of p53 (Figure 3E).

### Dominant Negative p53 Protects Renal Tubular Cells During Cold Storage/Rewarming

**Figure 4:**
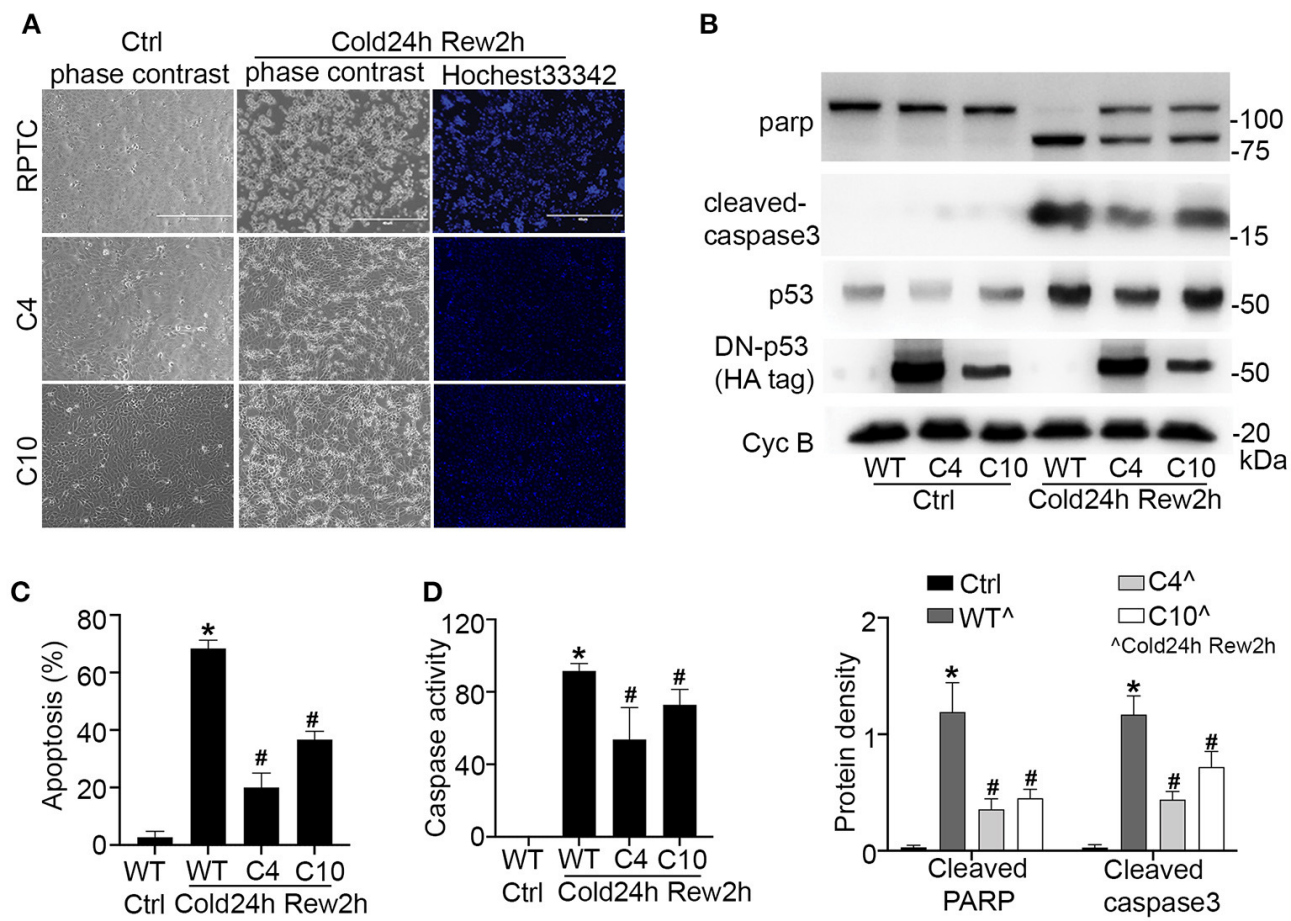
Dominant negative p53 protects renal tubular cells during cold storage/rewarming. Stably DN-p53 transfected cell clones (C4, C10) and wild type RPTCs were incubated in UW solution in 4°C for 24 h followed by rewarming in full medium in 37°C for 2 h. **(A)** Representative images of cellular and nuclear morphology. **(B)** Immunoblots analysis and quantification of PARP, cleaved caspase 3, p53 and DN-P53 marked by HA-tag with cyclophilin B as loading control. **(C)** Quantification of cell death. **(D)** Caspase activity. Data are expressed as mean ± SD (n≥ 5). *P < 0.05 vs. Control. ^#^*P* < 0.05 vs. WT Cold24h Rew2h.

To further verify the role of p53 in cold-storage rewarming injury, we tested RPTC cells that were stably transfected with dominant negative p53 (DN-P53) generated in our previous work (18). DN-P53 has a point mutation (V143A) that diminishes the DNA binding and gene transcriptional activity of p53. We compared regular/wild type (wt) RPTCs with two DN-p53 cell clones (C4, C10). The expression of HA-tagged DN-p53 in C4 and C10 cells (but not in wt cells) were confirmed by immunoblotting (Figure 4B). After cold-storage rewarming, C4 and C10 cells had 20 and 37% apoptosis, which were significantly lower than that of wt RPTCs (Figures 4A,C). C4 and C10 cells also had lower caspase activity (Figure 4D) and less cleaved PARP and caspase 3 (Figure 4B). Taken together, these results indicate that p53 plays an important role in cell injury and death in this cold storage/rewarming model.

### Pifithrin-α Protects Kidneys During Cold Storage/Transplantation

**Figure 5:**
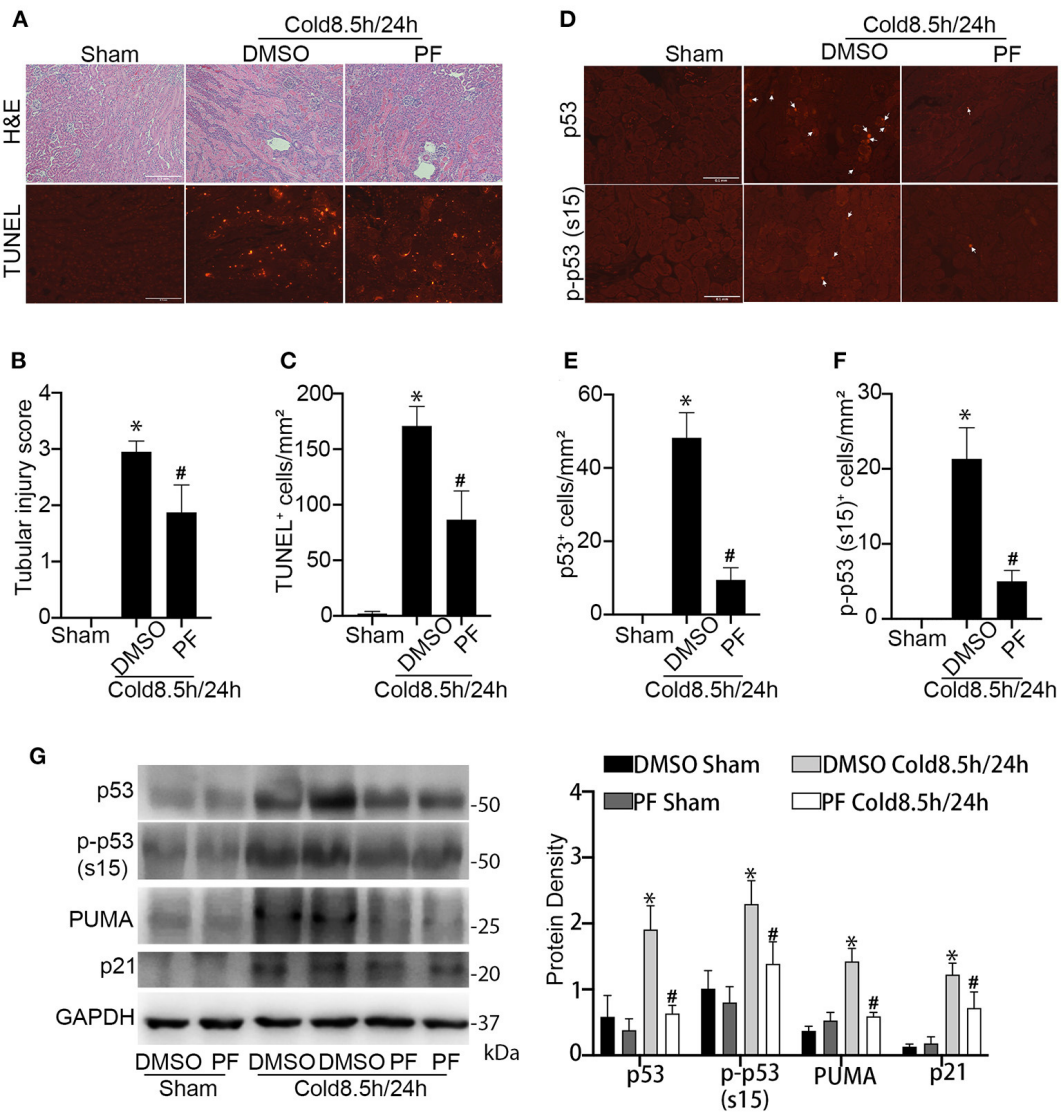
Pifithrin-α protects kidneys during cold storage/transplantation. 2.2 mg/kg pifithrin-α or the vehicle solution DMSO was injected intraperitoneally to donor mice. After overnight treatment, donor kidneys were collected for 8.5 h of cold storage in UW solution in the presence of 50 μM pifithrin-α or DMSO. After kidney transplantation surgery, 2.2 mg/kg pifithrin-α or DMSO was added to the abdomen cavity of recipient mice. The transplanted kidneys were collected at 24 h after transplantation. The contralateral kidneys of donor mice were used as sham control. **(A)** Representative images of H&E staining of renal histology (Scale bar, 0.2 mm) and TUNEL assay (Scale bar, 0.1 mm). **(B)** Tubular damage score. **(C)** Quantification of TUNEL positive cells in outer medulla and cortical tissues. **(D)** Representative image of p53 and p-p53 (s15) immunofluorescence. Scale bars, 0.1 mm. Arrow: positive staining. **(E,F)** Quantification of p53 and p-p53 (s15) positive cells in outer medulla and cortical tissues. Quantitative data are expressed as mean ± SD (n ≥ 4). *P < 0.05 vs. sham control, ^#^*P* < 0.05 vs. DMSO Cold 8.5 h/24 h. **(G)** Immunoblots analysis and quantification of p53, p-p53 (s15), p21 and PUMA with GAPDH as loading control.

We then determined the effect of pifithrin-α on kidney injury during cold storage/transplantation. To this end, the donor mice were pre-treated with pifithrin-α (drug administration detailed in materials and methods) to collect the left kidney for 8.5 h cold storage followed by transplantation for 24 h. The right kidney of donor mice without cold storage/transplantation was used as sham control. Pifithrin-α did not show effect on histology (data not shown) and protein expression in sham control. In histology analysis, cold storage/transplantation led to significant renal tubular damage (DMSO), which was reduced by pifithrin-α (PF). By semi-quantification, the tubular injury score was 3 in DMSO-treated group and 1.9 in pifithrin-α–treated mice (Figures 5A,B). Consistently, less tubular cell death was detected in pifithrin-α treated transplants, showing 171 TUNEL positive cells per mm^2^ tissue in DMSO group and 87 in pifithrin-α group (Figures 5A,C).

The inhibition of p53 by pifithrin-α was verified by immunofluorescence and immunoblot analysis. As shown in Figures 5D-F, kidney transplantation after 8.5 h of cold storage induced p53 accumulation and phosphorylation in many kidney cells, which was markedly reduced by pifithrin-α. Immunoblotting of p53, p-p53 (s15), p21 and PUMA further supported p53 inhibition by pifithrin-α in cold storage/transplantation (Figure 5G).

### Ablation of p53 From Proximal Tubules in Donor Kidneys Reduces Cold Storage/Transplantation Injury

**Figure 6:**
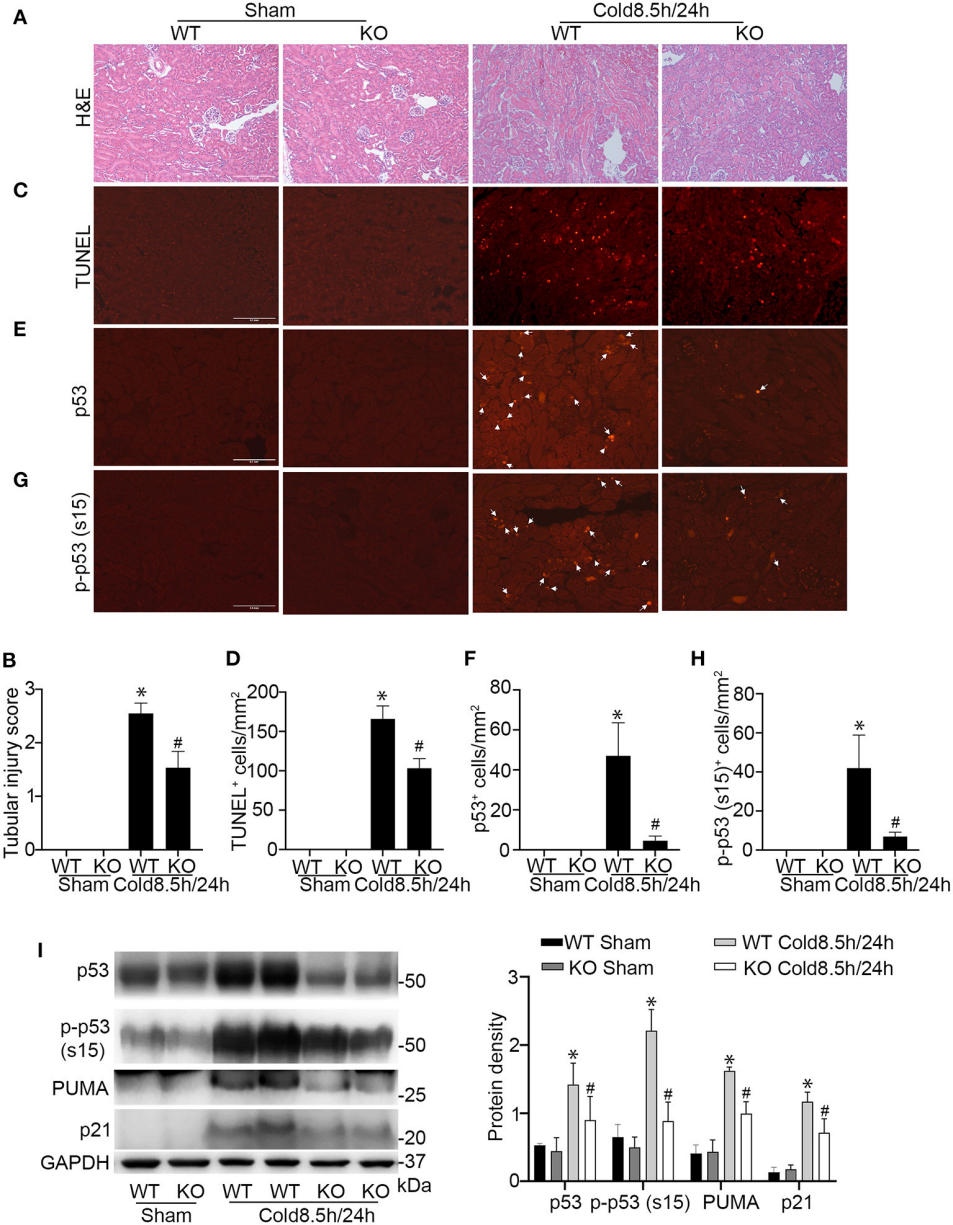
Ablation of p53 from proximal tubules in donor kidneys reduces cold-storage transplantation injury. Donor kidneys were collected from p53 proximal tubules knockout mice (KO) and their wild-type littermates (WT), stored in cold UW solution for 8.5 h, and transplanted into WT recipient mice. The transplanted kidneys were collected 24 h after transplantation for analysis. The contralateral kidney of donor was used as sham control. **(A)** Representative images of H&E staining. Scale bar, 0.2 mm. **(B)** Pathologic score of tubular damage. **(C)** Representative images of TUNEL assay. Scale bar, 0.1 mm. **(D)** Quantification of TUNEL positive cells in outer medulla and cortical tissues. **(E)** Representative image of p53 immunofluorescence staining. Scale bars, 0.1 mm. Arrow: positive staining. **(F)** Quantification of p53 positive cells in outer medulla and cortical tissues. **(G)** Representative image of p-p53 (s15) immunofluorescence staining. Scale bars, 0.1 mm. Arrow: positive staining. **(H)** Quantification of p-p53 (s15) positive cells in outer medulla and cortical tissues. Quantitative data are expressed as mean ± SD (n ≥ 4). *P < 0.05 vs. sham control, ^#^*P* < 0.05 vs. WT Cold 8.5 h/24 h. **(I)** Immunoblots analysis and quantification of p53, p-p53 (s15), PUMA and p21 with GAPDH as loading control.

To further establish the role of p53 in cold-storage transplantation injury, we took advantage of the conditional p53 knockout mouse model where p53 is specifically ablated from kidney proximal tubule cells. We isolated the kidneys from the p53 conditional knockout (KO) mice or their wild type littermates (WT) for 8.5 h of cold storage, and then transplanted into WT recipient mice for 24 h. As shown in Figures 6A,B, cold storage/transplantation induced significant tubular damage in the transplanted WT kidney mainly in outer stripe of outer medulla and partially in cortex with an injury score of ~2.5. In contrast, p53 KO graft showed a better preservation of the tubules at both sites, with overall injury score of ~1.5. Consistently, kidney cell death detected by TUNEL assay was ameliorated in KO transplants. In quantification, WT grafts had about 166 TUNEL-positive cells/mm^2^ tissue where 103 in KO kidney transplants (Figures 6C,D). Immunofluorescences and immunoblots of kidney cortex lysate showed massive p53 accumulation and phosphorylation in WT transplants along with target gene expression (Figures 6E–I), which was attenuated in KO grafts to demonstrate decent knock out efficiency in proximal tubules.

### p53 Inhibition Ameliorates Inflammation in Kidney Cold Storage/Transplantation

**Figure 7:**
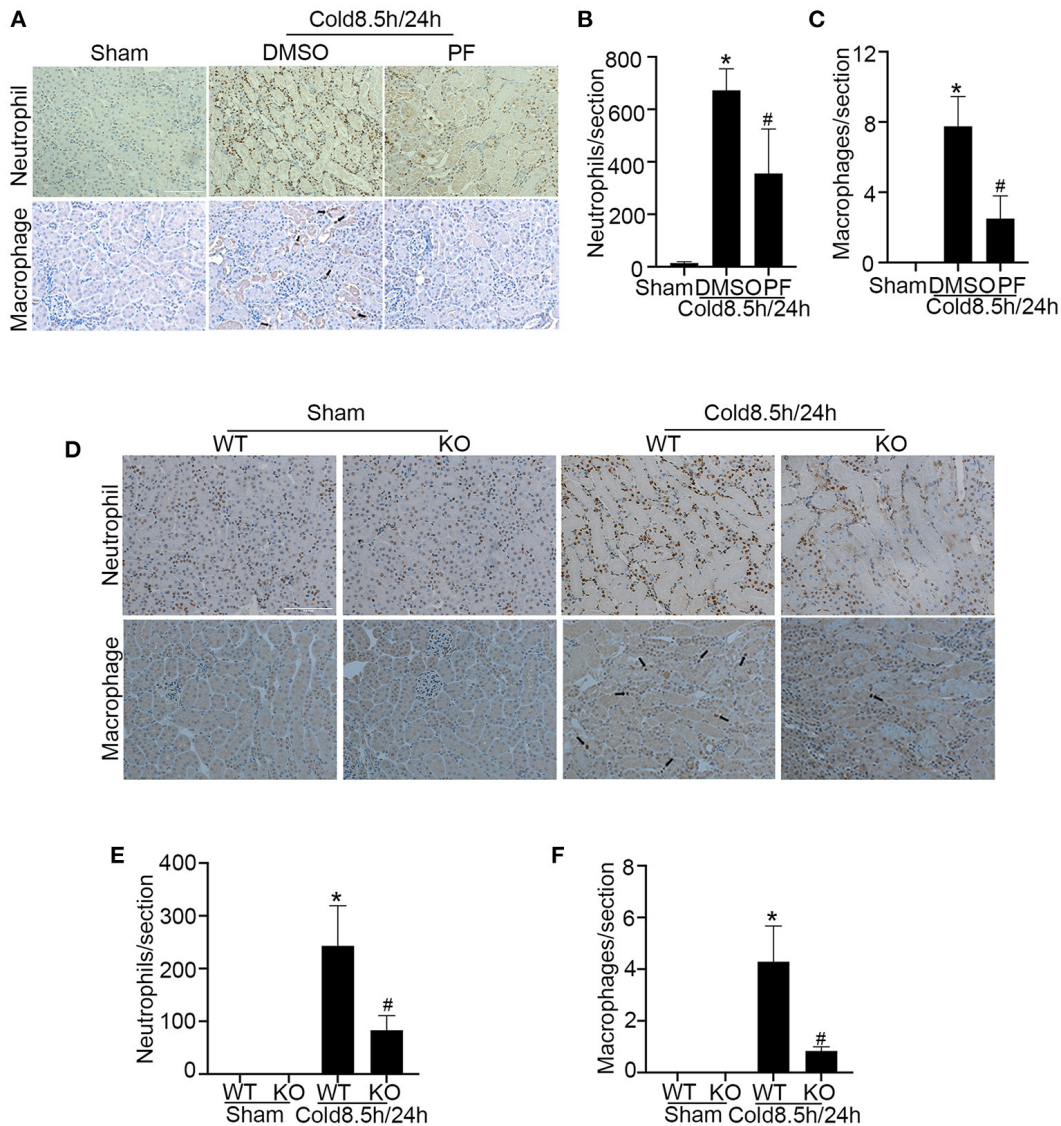
p53 inhibition ameliorates inflammation in kidney cold storage/transplantation. **(A–C)** Pifithrin-α or the vehicle solution DMSO were given to donor B6 mice overnight, during 8.5 h of cold storage, and immediately after transplantation as detailed in Figure 5. The transplanted kidneys were collected at 24 h after transplantation for analysis. **(A)** Immunohistochemistry of neutrophils and macrophages. Scale bar, 0.1 mm. Arrow: positive staining of macrophages. **(B)** Quantification of positive neutrophils per cross-sectional area. **(C)** Quantification of positive macrophages per cross-sectional area. **(D–F)** Kidneys were collected from p53 KO and their WT littermates, stored in cold UW solution for 8.5 h, and transplanted into WT recipient mice. The transplanted kidneys were collected 24 h after transplantation for analysis. **(D)** Immunohistochemistry of neutrophils and macrophages. Scale bar, 0.1 mm. Arrow: positive staining of macrophages. **(E)** Quantification of positive neutrophils per cross-sectional area. **(F)** Quantification of positive macrophages per cross-sectional area. Data are expressed as mean ± SD (n ≥ 4). *P < 0.05 vs. sham control, ^#^*P* < 0.05 vs. DMSO Cold 8.5 h/24 h or WT Cold 8.5 h/24 h.

In addition to tubular damage, kidney cold storage/transplantation is often associated with inflammation which contributes to both acute and chronic graft dysfunction (30). We analyzed inflammatory cell infiltration in transplanted kidneys after cold storage. As shown in Figure 7A, cold storage/transplantation induced the infiltration of numerous neutrophils and a few macrophages in kidney interstitial tissues, which was significantly suppressed by pifithrin-α (Figures 7B,C). Consistently, p53 ablation also suppressed the infiltration of neutrophils and macrophages during kidney cold storage/transplantation (Figures 7D–F). These results indicate that p53 may contribute to the inflammatory response in cold storage/transplantation by inducing tubular injury.

### Pifithrin-α Protects Against Kidney Injury and Dysfunction in Life-Supporting Grafts

**Figure 8:**
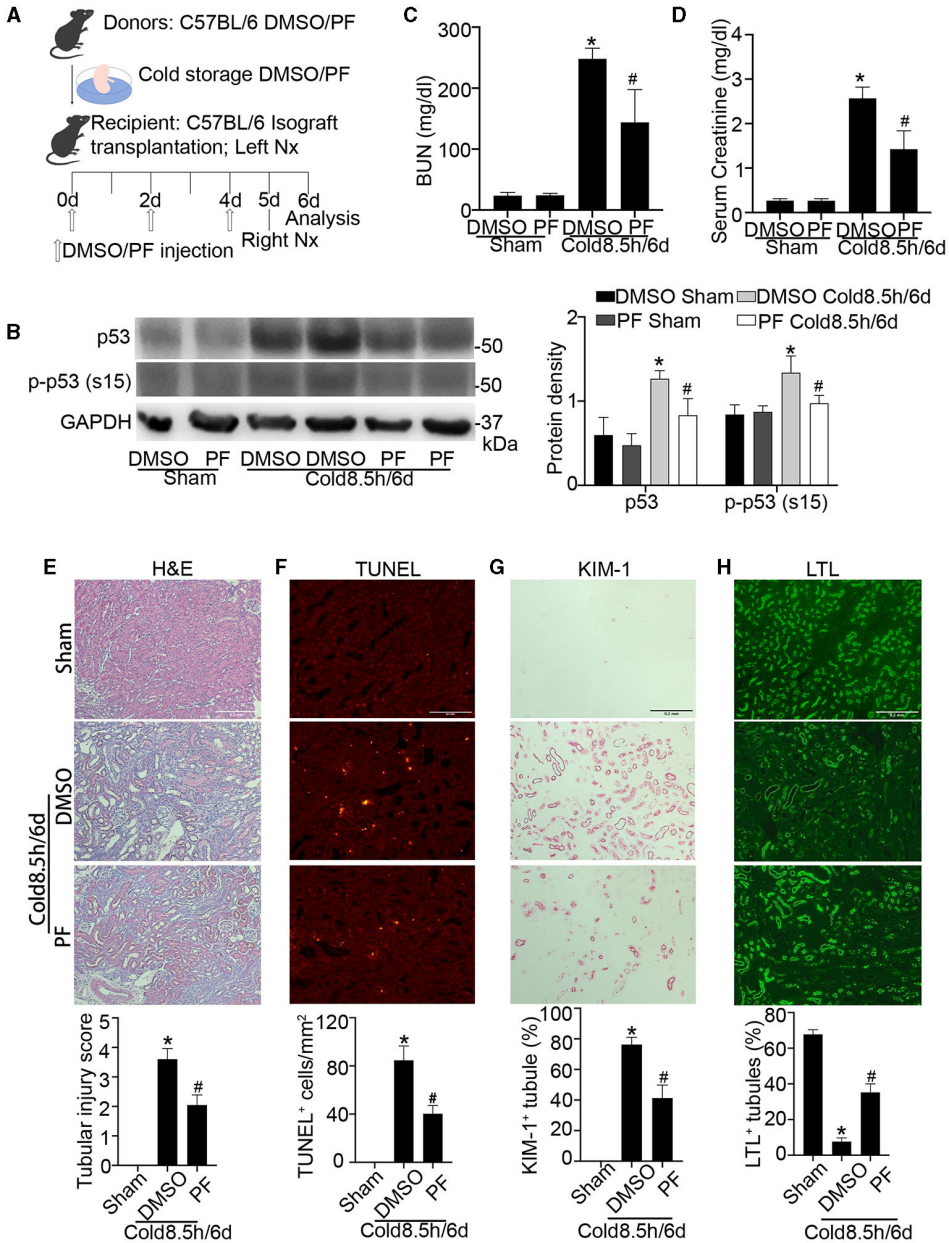
Pifithrin-α protects against kidney injury and dysfunction in life-supporting grafts. 2.2 mg/kg pifithrin-α or the vehicle solution DMSO was injected intraperitoneally to donor mice. After overnight treatment, donor kidneys were collected for 8.5 h of cold storage in UW solution with 50 μM pifithrin-α or DMSO. After kidney transplantation, 2.2 mg/kg pifithrin-α or DMSO was given three times to the recipient mice: immediately after surgery, on day 2 and day 4. On day 5 post-transplantation, the native kidney of the recipient mice was removed so that the transplanted kidney became the life-supporting kidney. Samples were collected on day 6 for analysis. The contralateral kidneys of donor mice were used as sham control. **(A)** Diagram depicting the experimental procedure. **(B)** Immunoblots analysis and quantification of p53 and p-p53 (s15) with GAPDH as loading control. **(C)** BUN measurement. **(D)** Serum creatinine measurement. **(E)** Representative images of H&E staining and tubular injury score. Scale bar, 0.2 mm. **(F)** Representative images of TUNEL assay and quantification of TUNEL positive cells. Scale bar, 0.1 mm. **(G)** Representative images of KIM-1 immunohistochemistry and percentage of KIM-1 positive tubules. Scale bar, 0.2 mm **(H)** Representative images of LTL staining and percentage of LTL positive staining tubules. Scale bar, 0.2 mm. Quantitative data are expressed as mean ± SD (n ≥ 4). *P < 0.05 vs. sham control, ^#^*P* < 0.05 vs. DMSO Cold 8.5 h/6 days.

To examine the therapeutic potential of p53 inhibition for transplanted kidneys, we established a life-supporting model, in which the recipient original kidney was removed on day 5 after transplantation so that the transplanted kidney became the life-supporting kidney as described in our recent work (9). DMSO or pifithrin-α was given before donor harvest, included in UW solution during cold storage and every other day after surgery as illustrated in Figure 8A (drug administration detailed in materials and methods). p53 inhibition by pifithrin-α in this experiment was verified by immunoblotting of p53 and p-p53 (s15) (Figure 8B). On day 6 after cold storage/transplantation, BUN and serum creatinine respectively increased to 248 mg/dl and 2.6 mg/dl in the mice receiving DMSO. Pifithrin-α significantly reduced BUN and serum creatinine to 144 mg/dl and 1.4 mg/dl (Figures 8C,D). In histological analysis, almost all tubules were injured with an injury score of 3.6 in DMSO group, whereas nearly half of renal tubules were preserved in mice receiving pifithrin-α with a tubular injury score of 2 (Figure 8E). Consistently, TUNEL assay showed that pifithrin-α reduced tubular cell death from 85 in DMSO group to 40 per mm^2^ tissue (Figure 8F). We further examined KIM-1 and LTL, which are known markers for kidney tubular injury and normal proximal tubules respectively (31). As shown in Figures 8G,H, pifithrin-α reduced the number of KIM-1 positive tubules while increasing the percentage of normal LTL staining tubules. Together, these results demonstrate the beneficial effects of pifithrin-α on cold storage/transplantation in life-supporting grafts.

## Discussion

Cold storage is commonly used for transplantation of kidneys from deceased donors based on its superiority in lowering metabolism and preserving viability (32). However, prolonged cold storage inevitably causes kidney injury and graft dysfunction, which are associated with lower donor utilization rate and poor outcome of renal transplants (33, 34). There is an urgent need to develop new and effective strategies to improve organ preservation, including the addition of renoprotective agents to the preservation solution and machine perfusion (35, 36). To identify such strategies, it is critical to elucidate the molecular and cellular events triggered in cold storage/transplantation that leads to injury and dysfunction of renal grafts. In this study, we found that p53 was activated during kidney cold storage/transplantation in mice and this activation correlated well with kidney tubular injury. p53 was also activated during cold storage/rewarming in RPTC cells. Functionally, pharmacological and genetic inhibition of p53 afforded protective effects in these models. Especially, the ablation of p53 from kidney proximal tubules reduced cold storage/transplantation kidney injury in mice, pinpointing proximal tubules cells as the main cell type of p53 action. The pharmacological inhibitor of p53, pifithrin-α, not only attenuated acute kidney injury during cold storage/transplantation but also improved the graft function later on when it became the life supporting kidney, suggesting the therapeutic potential of p53 inhibitors.

p53 has been implicated in the pathogenesis of both acute and chronic kidney diseases (15). Particularly relevant to this study is the observation that p53 participates in tubular cell injury and death during renal ischemia-reperfusion injury (IRI) or ischemic AKI (19). In cold storage/transplantation, the donor kidney experiences warm ischemia during organ procurement and vascular anastomosis; and cold ischemia during organ preservation, followed by reperfusion after transplantation to the recipient (37, 38). Therefore, renal IRI is a primary cause of acute and chronic nephropathies in kidney cold storage/transplantation. In this regard, our current study has further proved the pathogenic role of p53 by using models of kidney transplantation, one of the most relevant clinical conditions of renal IRI. Consistent with this study, Imamura et al. reported that systemic administration of siRNA for p53 ameliorates structural and functional damage in rat models of rat kidney transplantation (39). In renal IRI models, p53 in kidney proximal tubules (and not in other kidney tubules) plays a critical role in tubular cell death and renal functional loss (19, 20). Consistently, in the current study, we provided specific evidence that proximal tubule p53 play critical role in cold storage/transplantation injury in mice, which is convincing for its great similarity to human genome.

In our study, p53 was mainly activated in renal tubular cells during kidney cold storage/transplantation (Figures 1, 6). However, the mechanism of p53 activation under this condition remains unclear. A well-documented mechanism of p53 activation is DNA damage response, which includes a signaling cascade leading to the activation of upstream protein kinases to phosphorylate p53 (40, 41). Currently, it is unknown whether and to what extents DNA is damaged in kidney cold storage/transplantation, and whether the signaling pathway of DNA damage response is activated. Nonetheless, oxidative stress is associated with kidney cold storage/transplantation and, the beneficial effects of antioxidants, such as mitoQ (10, 28, 42), have been demonstrated for transplanted kidneys. Oxidative stress may lead to DNA oxidization, a known form of DNA damage, for the formation of oxidized nucleosides such as 8-oxo-2'-deoxyguanosine (8-oxo-dG). Therefore, oxidative stress is a likely cause of DNA damage response for p53 activation in kidney cold storage/transplantation.

The regulation of p53 is multifaceted and tissue/cell type specific (43). By using proximal tubule-specific p53 knockout model, we demonstrated the crucial role of p53 in proximal tubules during kidney transplantation with cold storage (Figure 6). Downstream of p53, both transcription-dependent and independent mechanisms may lead to nephrotoxicity (16, 18) and ischemic AKI (11, 19). In the current study, we examined down-stream genes of p53, such as p21 and PUMA. These genes were induced during kidney cold storage/transplantation, and the induction was attenuated by pifithrin-α and also by the ablation of p53 from proximal tubules (Figures 5, 6). In addition, tubular cell death during cold storage/rewarming was suppressed by dominant negative p53 that has a loss of transcriptional function mutation (18, 44). These results suggest that p53 mediates cold storage/transplantation kidney injury mainly by inducing pro-death genes through its transcriptional mechanism.

Inflammation is another pathological feature of, and pathogenic factor in, renal IRI and subsequent renal interstitial fibrosis (45). In the present study, we showed that cold storage/transplantation induced massive neutrophil infiltration but only a few scattered macrophages in 24 h (Figure 7). It is acknowledged that inflammatory cells especially neutrophils and associated cytokines play a pathogenic role in acute injury of kidney ischemia-reperfusion (46) However, the effect of pifithrin-α in inflammation and fibrosis is controversial from previous studies. It was reported that pifithrin-α exacerbated inflammation damage in rat (47) and mice (48) since inflammatory cell apoptosis may be suppressed. But knockout of p53 from kidney proximal tubules inhibited inflammation as the consequence of reduced tubular injury (19, 20). pharmacological and genetic blockade of p53 mitigated the infiltration of neutrophils and macrophages concomitantly with decreased tubular injury, indicating that reduced tubular damage led to the suppression of inflammation during kidney cold storage/transplantation. As for fibrosis, it was reported that pifithrin-α promoted renal fibrosis in rat model of ischemic AKI (47) although the mechanism is unclear. In contrast, it was also demonstrated that pifithrin-α attenuated fibrosis through facilitating G-M cell cycle progression after unilateral renal IRI in mice (49) and bilateral IRI in rats (50). Whether pifithrin-α can reduce renal fibrosis after kidney cold storage/transplantation warrants further investigation.

In conclusion, we have demonstrated an important role of p53 in kidney injury and dysfunction following cold storage/transplantation. In this regard, p53 in kidney proximal tubules is particularly important. Pharmacological inhibitors of p53, such as pifithrin-α, may have beneficial effects for improving the quality of donor kidneys from deceased donors and ultimately the outcome of renal transplants.

## Data Availability Statement

The original contributions presented in the study are included in the article/Supplementary Material, further inquiries can be directed to the corresponding author.

## Ethics Statement

The animal study was reviewed and approved by Institutional Animal Care and Use Committee of Charlie Norwood Veterans Affairs Medical Center.

## Author Contributions

XX and ZD designed experiments and analyzed results. XX and JZ performed experiments. JZ and GZ instructed murine kidney transplantation surgery. PEPCK-Cre mice were kept by ZM. DN-p53 RPTCs were constructed by ML. XX, JZ, and ZD wrote the manuscript. All authors contributed to the article and approved the submitted version.

## Funding

This work was supported partly by Merit Review Award I01 BX000319 from the Department of Veterans Affairs of USA and the grants 5R01DK058831 and 5R01DK087843 from the National Institutes of Health of USA. ZD is a recipient of Senior Research Career Scientist award from the Department of Veterans Affairs of USA.

## Conflict of Interest

The authors declare that the research was conducted in the absence of any commercial or financial relationships that could be construed as a potential conflict of interest.

## Publisher's Note

All claims expressed in this article are solely those of the authors and do not necessarily represent those of their affiliated organizations, or those of the publisher, the editors and the reviewers. Any product that may be evaluated in this article, or claim that may be made by its manufacturer, is not guaranteed or endorsed by the publisher.

## Abbreviations

AKI: acute kidney injury
BUN: blood urea nitrogen
CKD: chronic kidney disease
CNVAMC: Charlie Norwood Veterans Affairs Medical Center
Cyc B: cyclophilinB
DCD: donor of circulatory death
DGF: delayed graft failure
DMSO: dimethyl sulphoxide
ESRD: end stage renal disease
H&E: hematoxylin and eosin
IRI: Ischemia reperfusion injury
KIM-1: kidney injury molecular-1
KO: knock out
Nx: nephrectomy
PF: pifithrin alpha
PI: propidium iodide
p-p53 (s15): phosphor-p53 (Ser15)
PUMA: p53 upregulated modulator of apoptosis
RPTCs: rat kidney proximal tubule cells
TUNEL: terminal deoxynucleotidyl transferase-mediated dUTP nick end labeling
UW solution: university of Wisconsin solution
WT: wild type.
