# Supplementary figures and images for: Proximal Tubule p53 in Cold Storage/Transplantation-Associated Kidney Injury and Renal Graft Dysfunction
Source: Front Med (Lausanne). 2021 Oct 22;8:746346. doi: 10.3389/fmed.2021.746346 (PMC8569378; doi:10.3389/fmed.2021.746346)

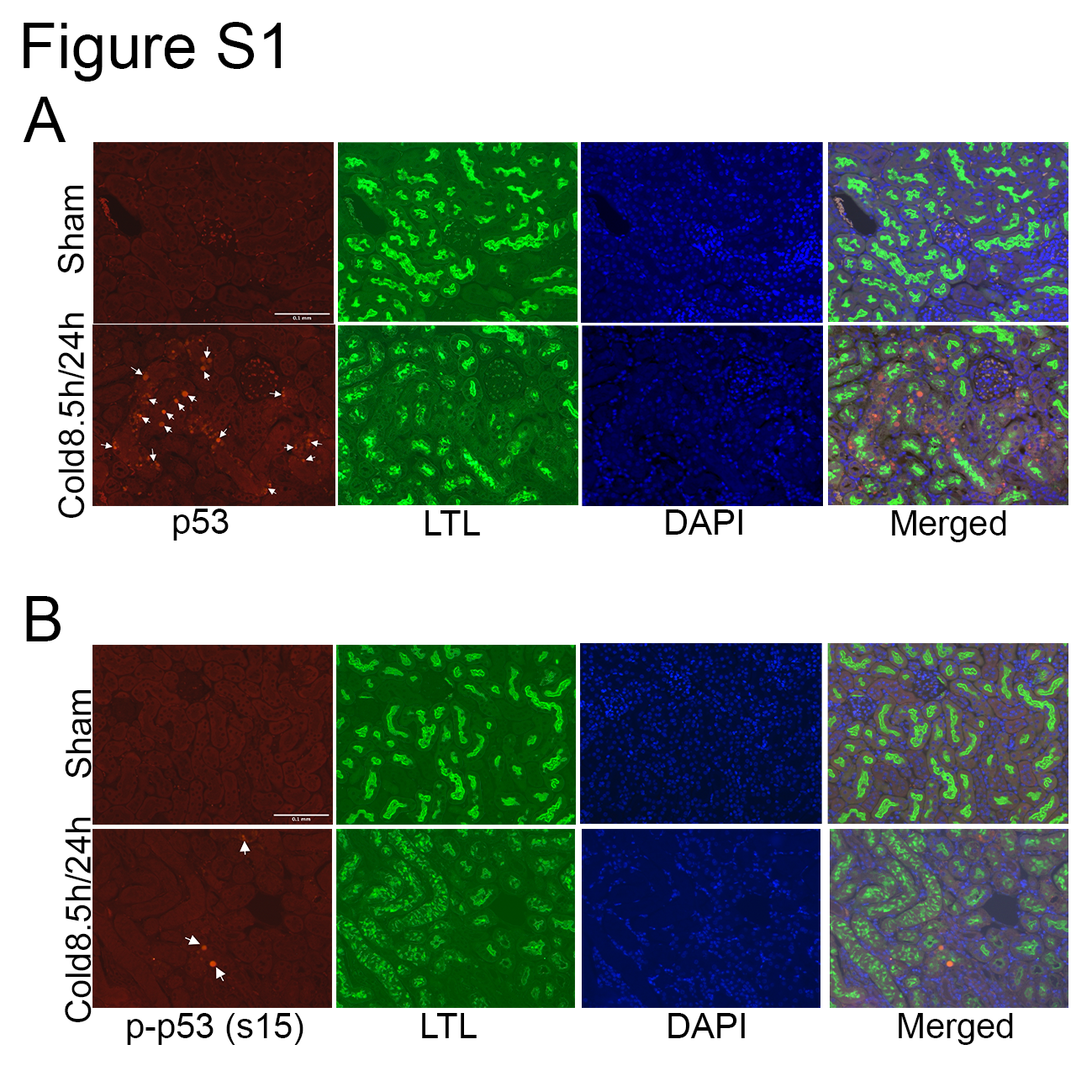

Supplement: Supplementary file 1 [file Image_1.TIF]

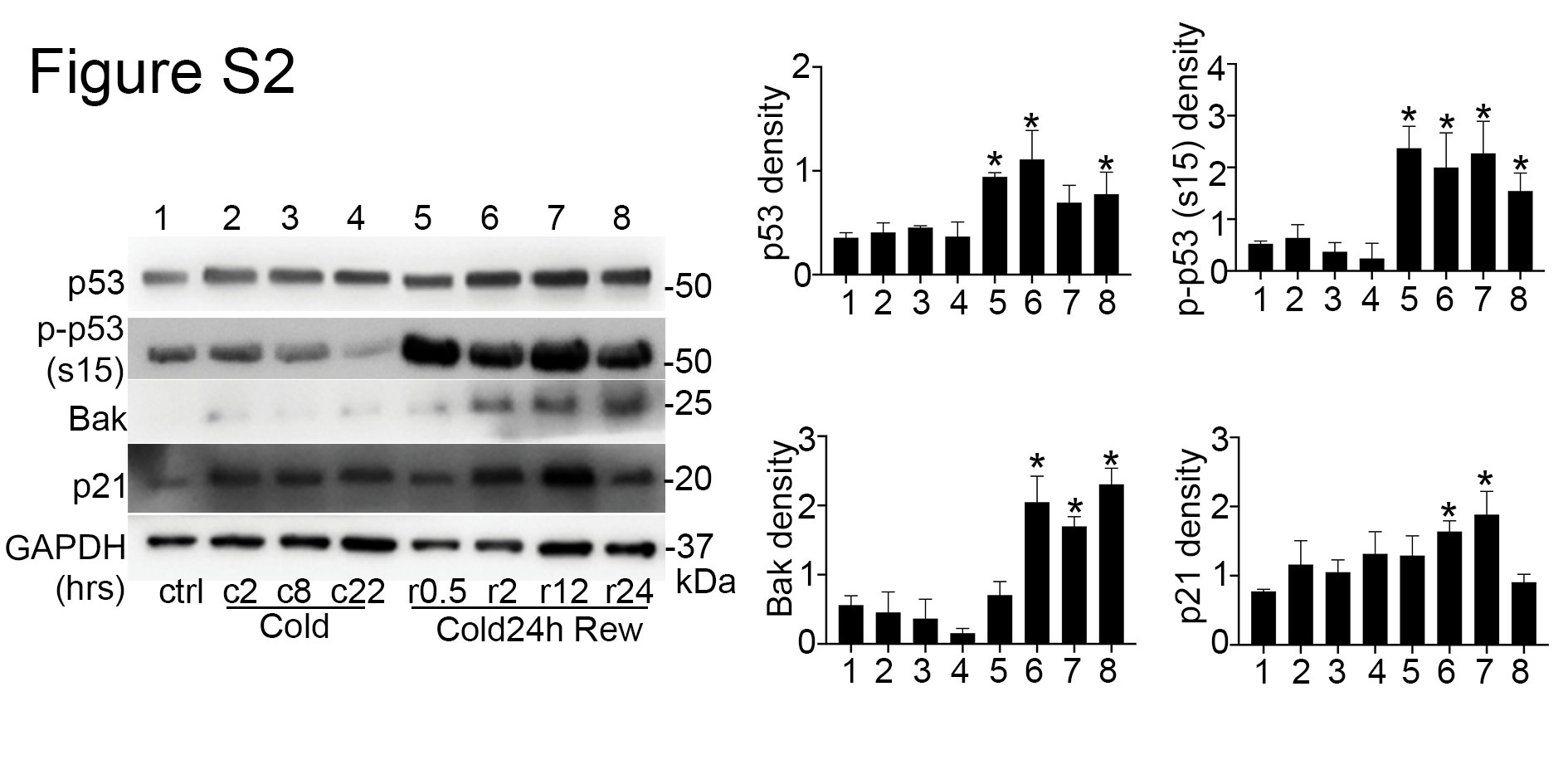

Supplement: Supplementary file 2 [file Image_2.TIF]
