# Supplementary material for: Proximal Tubule p53 in Cold Storage/Transplantation-Associated Kidney Injury and Renal Graft Dysfunction
Source: Front Med (Lausanne). 2021 Oct 22;8:746346. doi: 10.3389/fmed.2021.746346 (PMC8569378; doi:10.3389/fmed.2021.746346)
Supplement: Supplementary file 3 [file Data_Sheet_1.docx]

**Supplementary Materials**

**Supplementary Figure 1. p53 increases mainly in the cell nucleus of proximal tubules during kidney cold storage/transplantation.** The left kidney was isolated from the donor B6 mouse for 8.5h of cold storage and then transplanted to the recipient B6 mouse for 24h, right kidney of donor mouse without cold storage/kidney transplantation was used as sham control. (A, B) Representative image of p53 and p-p53 (s15) immunofluorescence staining. The tissues were co-stained with LTL to mark proximal tubules and with DAPI to mark cell nucleus. Merged images from 3 channels were showed. Scale bars=0.1mm. Arrow: positive staining.

**Supplementary Figure 2. p53 activation in RPTCs during cold storage/rewarming.** RPTCs were subjected to 2, 8, or 22h of cold storage in UW solution only, or 24h cold storage with 0.5, 2, 12, 24h of rewarming in culture medium. Cell lysate was collected for immunoblot analysis and quantification of p53, p-p53 (s15), p21 and Bak with GAPDH as loading control.
